# Effect of team-based learning on critical thinking: A quasi-experimental study

**DOI:** 10.12669/pjms.38.8.6146

**Published:** 2022

**Authors:** Muhammad Asif Zeb, Usman Mahboob, Neelofar Shaheen

**Affiliations:** 1 Muhammad Asif Zeb, BSc. MLT, MSc Hematology, MHPE. Lecturer, Institute of Paramedical Sciences, Khyber Medical University, Peshawar, Pakistan; 2 Usman Mahboob, MBBS, MPH, FHEA (UK), DHPE (UK), Fellow FAIMER (USA) Associate Professor, Institute of Health Professions Education (IHPE&R), Khyber Medical University, Peshawar, Pakistan; 3 Neelofar Shaheen, MBBS, MHPE, PhD Scholar, Assistant Professor, Department of Health Professions Education & Research (DHPE&R), Peshawar Medical College, Peshawar, KPK, Pakistan, Riphah International University, Islamabad, Pakistan

**Keywords:** Team-Based Learning, Critical Thinking, Active Learning, Students, Paramedics, Nursing

## Abstract

**Objectives:**

To determine the effect of Team-based learning (TBL) on the critical thinking of health professions students.

**Methods:**

This quasi-experimental study, was done in paramedical and nursing sciences institutes using the convenience sampling technique. Students included were enrolled in the haematology course of paramedic institute from January to December 2020 and adult health course of the nursing institute of Khyber Medical University. Six dimensions of critical thinking (CT) of the students before and after TBL were determined using the critical thinking disposition inventory.

**Results:**

The study participants included 89 students, comprising 58 students from the paramedic’s institute and 31 from the nursing institute; 67 (75.28%) males and 22 (24.71%) females. The overall pre-test score of CT was 257.46 ± 21.73, and the post-test score was 274.55 ± 19.36, which was statistically significant *(p-*value = 0.000). The pre-test score of six dimensions, namely, analyticity, inquisitiveness, systematicity, truth-seeking, self-confidence, and open-mindedness was 41.35 ± 5.15, 44.73 ± 4.77, 41.12±6.87, 43.17± 5.19, 44.94±6.03, 42.38 ± 5.32 respectively, whereas the post-test scores were 44.57± 5.28, 47.11 ± 4.69, 46.12± 5.54, 45.77 ± 5.05, 47.58 ± 5.65, 43.56 ± 4.56 correspondingly. Analyticity (*p*=.000), inquisitiveness (*p*=.000), systematicity (*p*=.000), truth-seeking (*p*=.000) and self-confidence (*p*=.000) were statistically significant. However, open-mindedness was statistically insignificant (*p*=.074).

**Conclusion:**

TBL improves five out of six dimensions of students’ critical thinking. Besides its established evidence to increase knowledge, TBL can also be used as a teaching methodology for enhancing students’ critical thinking.

## INTRODUCTION

Over the last decade, various teaching instructions have been introduced to shift the learning paradigm from passive to active learning and improve students’ critical thinking (CT).^1^ Passive learning is essentially teacher-centred, wherein information is transferred from the teachers to the students and is based on rote memorisation. In contrast, active learning is student-centred that actively engages students.^2^

Team-based learning (TBL) is one of the active teaching strategies specially designed for a large group of students.^3^ It provides an opportunity for the students to apply their knowledge interactively. Students work together in groups, and support and motivate each other in the learning process.^4^ TBL is done in four phases. The students are provided assignments either from literature or a short lecture and videos in the preparatory phase. The students work at home and then apply their knowledge in class with their teammates. Upon arrival into class, an Individual Readiness Assurance Test (iRAT) is conducted, consisting of 10 to 20 multiple-choice questions (MCQs). The students solve the same MCQS of iRAT in a team, and after in-depth discussion and debate, understand the assigned topic entirely.^5^ In the application phase, the students are introduced to a problem related to real-life; they discuss it in groups, support each other and collect relevant information to solve the problem. In the end, the instructor provides feedback to the students based on their performance.^1^

The literature has reported that TBL improves knowledge compared to PBL, and CBL and also involves active teaching techniques.^6^ One of the advantages of active teaching techniques is improving critical thinking.^3^ Critical thinking (CT) is at the heart of knowledge-seeking by the students as the changing times need a highly capable human resource, which institutions need to produce. TBL is one of the strategies that can have a pivotal role in developing CT among students. There is conflicting evidence of whether TBL improves critical thinking or not.^7^ A previous study on TBL showed an increase in one dimension of critical thinking.^8^ However, the TBL sessions in this study were not standardised and the mini-lectures were delivered at the beginning of the TBL session.^8^ Critical thinking has multiple dimensions and it needs to be established whether TBL influences all of these dimensions.^7^ Our study aimed to determine the effects of TBL on different dimensions of critical thinking of nursing and paramedical students.

## METHODS

**Fig.1:**
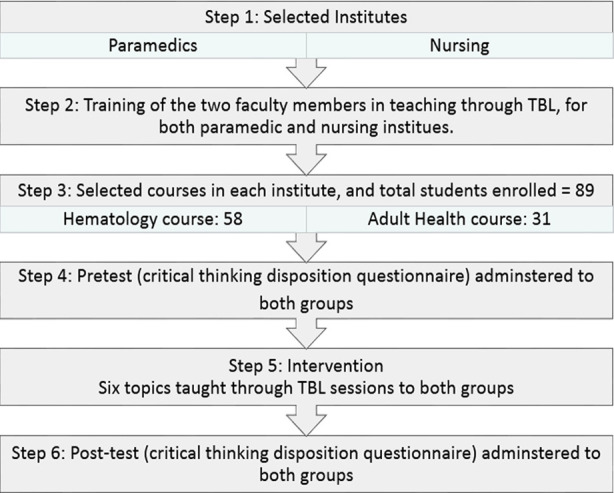
Flow diagram for the steps of quasi-experimental study to determine the effect of Team-based Learning (TBL) on critical thinking.

A quasi-experimental pre-test post-test study design was employed at the Institute of Paramedical Sciences (IPMS) and the Institute of Nursing Sciences (INS), Khyber Medical University, from January to December 2020. We could not find specific guidelines for quasi-experimental studies, so we followed the CONSORT guidelines except the randomisation and control group (Fig.1).^9^ Students enrolled, during the study time, in the haematology course at IPMS and adult health course at INS were included, and repeater students were excluded from the study. The convenience sampling technique was used because of the limited number of available students. Informed consent was taken from the students before the study. The Ethics Committee of the Khyber Medical University approved the study (ref: DIR/KMU-EB/ET/000692). The administrative permission for this study was also taken from the heads of the institutions. Two faculty members were trained in conducting TBL sessions of the students through a training workshop. The faculty members then conducted two TBL sessions with the students under supervision.

Moreover, we prepared tutor guides for the training of the faculty members. The tutor guide consisted of guidelines on TBL sessions. Also, the students were informed about the steps of TBL and that their assessments will not be affected as the exact topics will be taught again through regular teaching sessions after the study. Six TBL sessions were conducted; each session took two hours and 30-minutes. Before running the TBL session, a critical thinking disposition questionnaire was distributed.

The critical thinking disposition questionnaire consisted of 51 items on a 6-point Likert scale that is Strongly Disagree (SD), Disagree (DG), Partly Disagree (PD), Partly Agree (PA), Agree (AG), and Strongly Agree (SA). The questionnaire covers six dimensions of CT: inquisitiveness, analyticity, open-mindedness, self-confidence, systematicity, and truth-seeking. The total score of the scale ranged from 60 to 360. Individuals with scores below 240 were considered to have low critical thinking skills, while individuals with more than 300 were supposed to have higher critical thinking skills.^10^

The data were analyzed using SPSS version 22.0. For categorical variables such as gender and institutions, frequency tables were used. Paired sample T-test was applied at a 95% confidence interval before and after TBL intervention.

## RESULTS

Eighty-nine undergraduate students participated in this study, 58 from IPMS and 31 from INS. Amongst them, 67 (75.28%) were males, and 22 (24.71%) were females. The critical thinking score was determined by combining all the components of critical thinking. The mean score of pre-test critical thinking was 257.46±21.73, while post-critical thinking was 274.55±19.36. The significance of the scores is mentioned in Table-I. The mean test score and standard deviation of pre-test and post-test score for inquisitiveness were 44.73 ± 4.77, 47.11 ± 4.69; for analyticity pre-test and the post-test score were 41.35 ± 5.15 and 44.57 ± 5.28; for open-mindedness, the score was 42.3839 ± 5.32 and 43.56 ± 4.56 respectively. The self-confidence means pre-test and the post-test score were 44.94 ± 6.03 and 47.58 ± 5.65 respectively; for truth-seeking, the mean pre-test and post-test score were 43.1781 ± 5.19 and 45.77 ± 5.054; whereas for systematicity pre-test and the post-test score was 41.12 ± 6.87 and 46.1231 ± 5.54 respectively (Table-I).

**Table-I:** Combined critical thinking scores of paramedics and nursing students.

Critical thinking dimensions pre-test – post-test scores	Mean	Sth. deviation	P-value
Pre-test inquisitiveness score - Post-test inquisitiveness score	2.38	5.64	.000
Pre-test analyticity score - Post-test analyticity score	3.21	5.96	.000
Pre-test open-mindedness score - Post-test open-mindedness score	1.17	6.15	.074
Pre-test self-confidence score - Post-test self-confidence score	2.64	6.24	.000
Pre-test truth-seeking score - Post-test truth-seeking score	2.60	6.41	.000
Pre-test systematicity score - Post-test systematicity score	4.99	7.98	.000
Pre-test critical thinking score - Post-test critical thinking score	17.09	25.69	.000

We also separately determined the two institutes, IPMS and INS students, on the sub-dimensions CT scores (Table-II). Separately in both the institutes, only open-mindedness was statistically insignificant.

**Table-II:** Critical thinking score of paramedics and nursing students.

Critical thinking dimensions pre-test – post-test scores	Paramedic Students (Haematology course)			Nursing Students (Adult health course)		

	Mean	Sth. deviation	P-value	Mean	Sth. deviation	P-value
Pre-test inquisitiveness score - Post-test inquisitiveness score	-3.07	5.62	.000	-4.84	8.22	.003
Pre-test analyticity score - Post-test analyticity score	-3.5	5.61	.000	-2.61	6.64	.036
Pre-test open-mindedness score - Post-test open-mindedness score	-1.14	5.65	.128	-1.24	7.09	.338
Pre-test self-confidence score - Post-test self-confidence score	-3.13	5.75	.000	-4.10	8.59	.012
Pre-test truth-seeking score - Post-test truth-seeking score	-2.83	6.18	.001	-2.67	6.32	.025
Pre-test systematicity score - Post-test systematicity score	-5.05	8.02	.000	-4.89	8.03	.002
Pre-test critical thinking score - Post-test critical thinking score	-19.04	23.79	.000	-13.42	28.98	.015

## DISCUSSION

The present study reports that TBL positively affects students’ critical thinking. The data revealed a statistical difference between pre-test and post-test critical thinking scores. The post-test critical test’s mean was higher than the pre-test critical thinking score, which is statistically significant as the *p*-value is less than .05 (0.000). It is evident from this study that after TBL, significant improvement occurred in the students’ critical thinking. Our results are similar to a previous study.^11^ TBL is an effective teaching method for collaborative learning and augments critical thinking.^12^ In collaborative learning, the students interact with each other, ask questions, and help each other without fear and hesitation. This collaborative learning in TBL is also perceived to improve academic performance.^13^,^14^ Moreover, solving challenging problems and application of knowledge may also lead to improved critical thinking.^15^

Critical thinking has six dimensions: inquisitiveness, analyticity, open-mindedness, self-confidence, systematicity, and truth-seeking. Inquisitiveness, analyticity, self-confidence, systematicity, and the truth-seeking result are statistically significant as the p-value is less than .05 (0.00). During TBL, the students gather relevant information to solve the assigned problem, discuss it in groups, ask questions, and take help from their teacher, enhancing their inquisitive and problem-solving abilities.^16^,^17^ In addition to this, during group discussion, the peers and the facilitator encourage the struggling students, which motivates the students and imparts confidence to them.^18^ Similarly, receiving feedback from both the peers and the teacher also helps build the students’ confidence.^19^ A study has reported that the confidence level of the students enhanced after TBL, which supports our finding that students’ self-confidence improved after TBL.^3^ Our result showed that TBL is a systematic approach to learning. Studies have reported that TBL enhances the students’ study habits and makes them responsible for their learning, favouring our results.^20^ Furthermore, starting from self-study in the preparatory phase till receiving feedback from their teachers, students systematically seek relevant information for solving real-life-related problems. Thus, TBL influences the ability of systematicity and the truth-seeking dimension of critical thinking.^21^

Open-mindedness is another dimension of critical thinking which is statistically not significant as the p-value is more than .05 (.074) at a 95% confidence interval. Our results show that TBL did not significantly improve the students’ open-mindedness. The educational system in our province is focused on traditional teaching methods, so shifting the students from traditional to active teaching methodology will require time.

The strength of our study is that it is done at two institutes, paramedical and nursing, and can be extended to other health sciences such as medicine, dentistry, physiotherapy, pharmacy, and allied health sciences. This study provides a base that TBL is a valuable mode of instruction for enhancing the critical thinking of nursing and paramedic students.

### Limitations:

One of the limitations of our study is that it shows the quantitative perspective of the difference in critical thinking, but not how does TBL improve CT? The other limitation of this study was having only two institutes: IPMS and INS, which are the constituent institutes of the Khyber Medical University. Hence, we recommend qualitative research to determine how TBL improves the students’ critical thinking and what factors foster critical thinking? The third limitation was that only one faculty member taught through TBL in each institute. We do not know if it was the teaching skills of these individual faculty members or the teaching method that improved students’ critical thinking of students? Fourth, a parallel active teaching method such as CBL or PBL can be used as a comparison group to control the Hawthorne effect, that is, if the students felt motivated due to a new teaching method, thus leading to improvement in critical thinking or if TBL improves critical thinking? Therefore, randomised control trials with comparable active teaching methods should be done to establish the effect of TBL on CT.

## CONCLUSION

TBL as a teaching methodology can improve the critical thinking of paramedic and nursing students. Through TBL, students’ analytical thinking improves, and the students systematically engage in collaborative learning. Additionally, the students become more inquisitive and keener to catch on to relevant information for solving complex problems.

### Author’s Contribution:

**MAZ:** Conceptualization, literature search, data collection, data analysis. Writing the manuscript and final editing of the manuscript. **UM:** Conceptualization, literature search, developed methodology, analysed data, critical revision manuscripts and final editing.

**NS:** Conceptualization, developed methodology, critical revision of all drafts, and final manuscript editing. Approval of the final manuscript. *All authors have read and approved, agree to be accountable for all aspects of the work in ensuring that questions related to the accuracy or integrity of any part of the work are appropriately investigated and resolved.
